# Case Report: Bowen’s disease treated with PD-1 inhibitor and chemotherapy

**DOI:** 10.3389/fonc.2025.1654431

**Published:** 2025-09-25

**Authors:** Xiaohui Xie, Qinyang Chen, Jian Zhang, Xiaodong Peng

**Affiliations:** ^1^ Department of Oncology, Chendu Second People’s Hospital, Chengdu, China; ^2^ Tha Academy of Chinese Health Risks, West China Hospital, Sichuan University, Chengdu, China

**Keywords:** Bowen’s disease, PD-1 inhibitor, chemotherapy, 5-fluorouracil, cutaneous squamous cell carcinoma

## Abstract

A 51-year-old man with perianal Bowen’s diseases, treated with tislelizumab and capecitabine chemotherapy, the tumor was significantly reduced and Improved quality of life.

## Background

Primary cutaneous malignancies are among the most common cancer types globally, with Bowen’s disease having an age-standardized incidence rate of 5.8% (1). Bowen’s disease, also known as squamous cell carcinoma *in situ* of the skin, typically affects the epidermal layer without often invading deeper skin tissues. Characteristically, it presents as a single, well-demarcated, irregular red patch in sun-exposed areas such as the head, neck, and extremities (2). However, in this particular case, the lesion occurred peri-anally, an area typically shielded from sunlight, and it exhibited an exceedingly rare, massive, cauliflower-like growth pattern.

## Treatment

**Figure 1:**
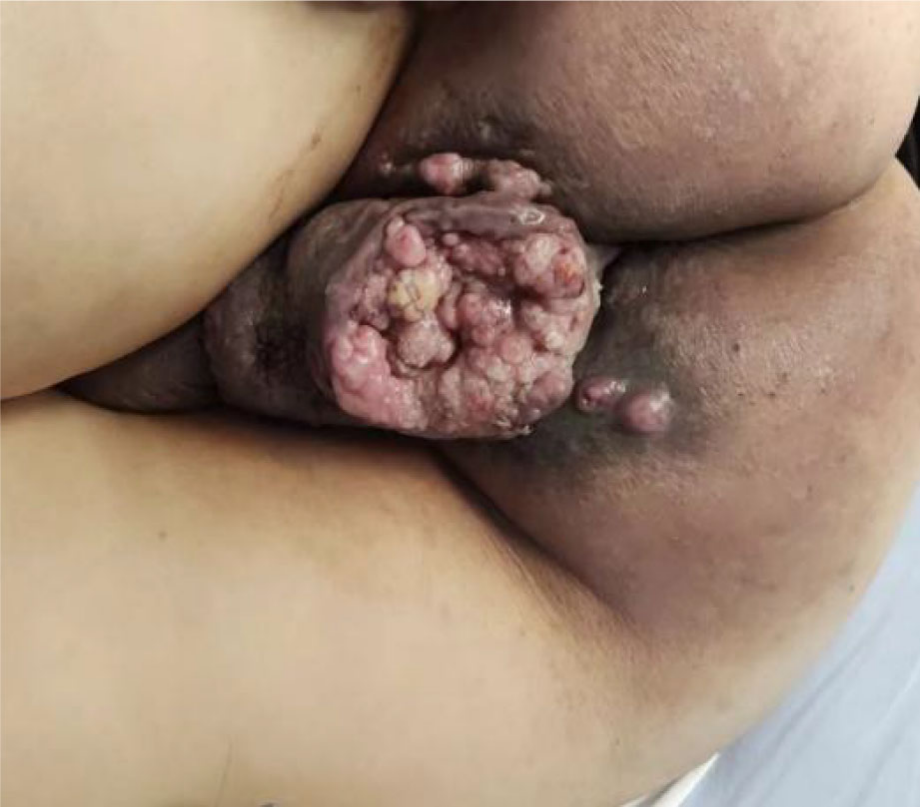
Pre-treatment appearance.

**Figure 2:**
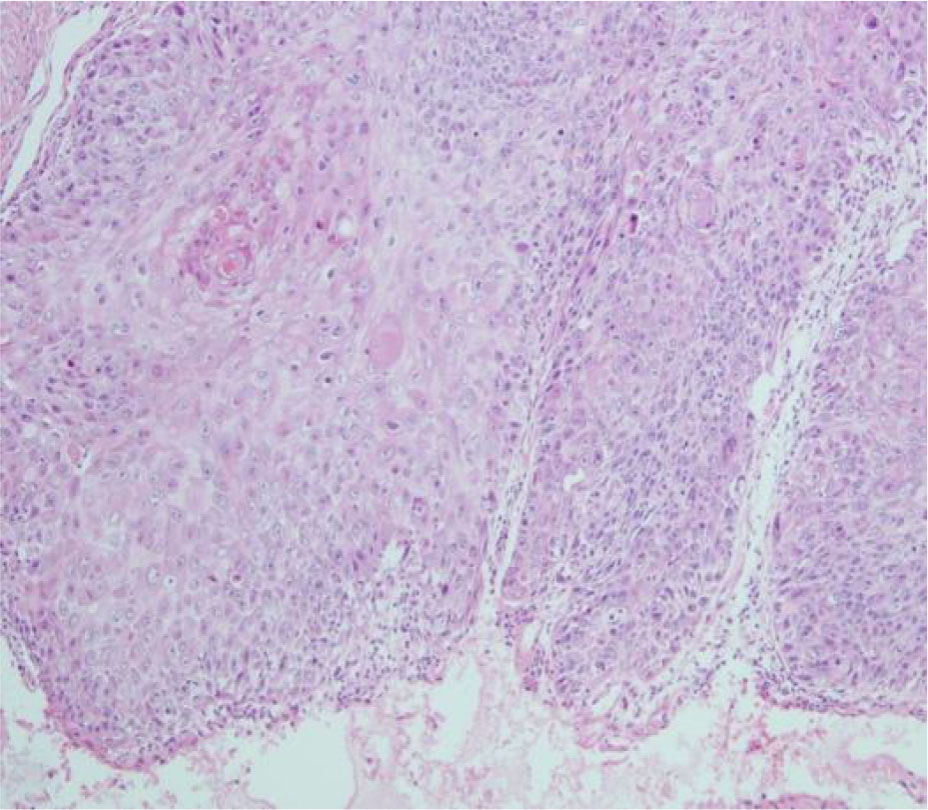
Pathological section.

**Figure 3:**
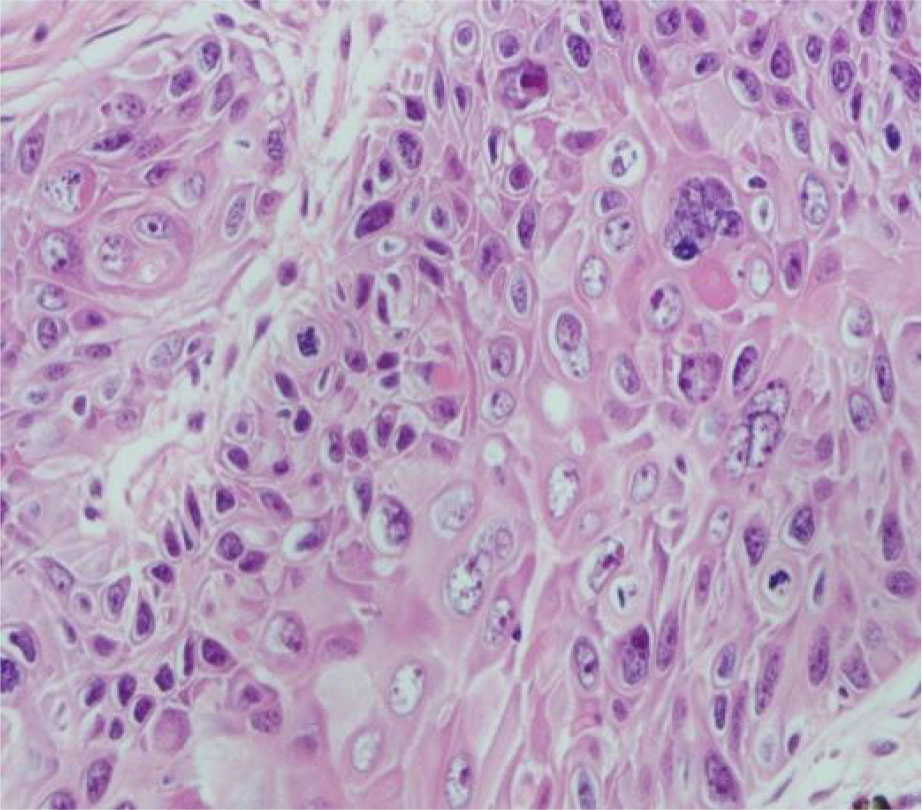
Pathological section.

**Figure 4:**
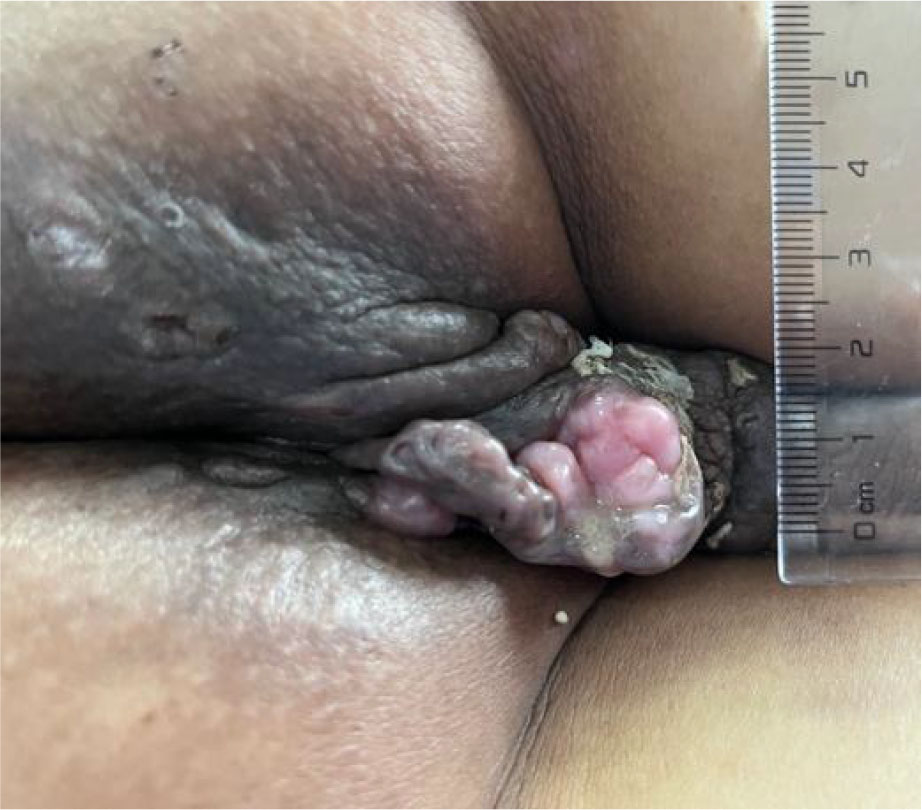
Post-treatment appearance after two treatment cycles.

A 51-year-old middle-aged man inadvertently noticed a soybean-sized swelling on the perianal skin, without obvious symptoms such as itching, pain, or bleeding and discharge. Three months later, the swelling gradually increased in size, accompanied by itching and discharge (**Figure 1**). A pathological biopsy revealed “perianal” Bowen’s disease (**Figures 2**, **3**), with suspected focal infiltration. After consultation with a surgical team, it was determined that the patient’s perianal tumor had grown significantly, involving the scrotum and perineum, with poor mobility, making radical resection impossible. Following departmental discussion, the patient was treated with tislelizumab and capecitabine chemotherapy, along with topical application of fluorouracil ointment on the tumor. After two cycles of treatment, the tumor was significantly reduced, and the third cycle of treatment was continued (**Figure 4**). However, the patient did not proceed with the fourth cycle of treatment due to personal reasons. Subsequent follow-ups have shown that the patient has survived for two years since then.

## Discussion

The exact etiology of Bowen’s disease remains unclear, but research has demonstrated that chronic exposure to ultraviolet radiation, arsenic exposure, various strains of HPV, chemical carcinogens, immunosuppression, and chronic irritation are considered to be some of the risk factors contributing to the development of Bowen’s disease (3). In this case, the patient experienced rapid and substantial growth of the lesion, which was also attributed to long-term scratching due to perianal itching, confirming that prolonged chronic irritation is one of the significant risk factors for the development of Bowen’s disease.

This is the first instance of treating Bowen’s disease with a combination of immune checkpoint inhibitors and 5-fluorouracil. The patient was a 51-year-old single man without employment or a stable income, so his treatment budget was tightly constrained. The tumor had grown to an exceptionally large size, infiltrating the scrotum, perianal area, and perineum, exhibiting poor mobility. Therefore, radical resection was impossible and any local modality was expected to yield only limited benefit. After multidisciplinary discussion, systemic therapy was chosen.Guidelines list the recommended options for inoperable Bowen’s disease in the following order: photodynamic therapy (PDT, grade A), topical fluorouracil, and cryotherapy (4). Although PDT is the preferred first-line modality, its efficacy is constrained by factors such as photosensitizer type, limited depth of cytotoxic effect, tumor hypoxia, and technical complexity; moreover, the shallow tissue penetration of light markedly reduces its activity against deeper tumor components (5).Numerous studies and guidelines have affirmed the role of 5-fluorouracil in squamous cell carcinoma (6, 7). The programmed cell death protein 1 (PD-1) inhibitors are monoclonal antibodies that block the interaction between PD-1 and its ligands, thereby restoring the immune system’s ability to attack tumor cells. They have become a cornerstone of immunotherapy for multiple advanced solid and hematologic malignancies (8). PD-1 inhibitor cemiplimab has been approved as a first-line treatment for patients with metastatic or locally advanced cutaneous squamous cell carcinoma (cSCC) (9), but considering the accessibility and cost of the medication, we ultimately opted for another PD-1 inhibitor, tislelizumab, in combination with 5-fluorouracil for treatment. Historical data indicate that topical fluorouracil is less effective than surgery and slightly inferior to photodynamic therapy, while robust evidence for immunotherapy in Bowen’s disease is still lacking. Nevertheless, for extensive lesions it represents a novel strategy worth investigating and may signal a future direction. In this case, evaluation after two cycles revealed marked tumor shrinkage, an outcome that is both encouraging and promising.

## Conclusion

In the era of immunotherapy, the combination of PD-1 inhibitors with 5-fluorouracil has proven effective in treating patients with Bowen’s disease who are not suitable candidates for local therapies, offering a systemic treatment approach for future management of patients with extensive Bowen’s disease.

## Data Availability

The raw data supporting the conclusions of this article will be made available by the authors, without undue reservation.
